# Containment Liner Plate Void Defect Detection Technique Using Phased Array Ultrasonic Testing and Acoustic Resonance Method

**DOI:** 10.3390/ma15041330

**Published:** 2022-02-11

**Authors:** Yun-Taek Yeom, Yeong-Won Choi, Hak-Joon Kim, Hun-Hee Kim, Jae-Suk Park, Sung-Woo Ryu, Sung-Jin Song

**Affiliations:** 1 School of Mechanical Engineering, Sungkyunkwan University, Suwon 16419, Korea; yeomtaek7@skku.edu (Y.-T.Y.); 0won@skku.edu (Y.-W.C.); sjsong@skku.edu (S.-J.S.); 2 Doosan Heavy Industries and Construction Co., Changwon 51711, Korea; hunhee1.kim@doosan.com (H.-H.K.); jaeseok1.park@doosan.com (J.-S.P.); 3 EnesG, Daejeon 34026, Korea; sungwoo.ryu@enesg.co.kr

**Keywords:** containment liner plate, PAUT, ARM, signal mapping, void defect

## Abstract

The CLP (containment liner plate) of a nuclear power plant protects the internal system from the external environment and sudden changes in internal pressure or temperature, and it is a structure that blocks and protects radioactive materials leaking inside and outside in the event of a nuclear accident and is composed of a liner plate, reinforcing bars, tendons, and concrete. Recently, corrosion on the rear side of the liner plate and concrete voids has emerged as a severe defect in nuclear power plants across South Korea. Therefore, in this study, we proposed a new inspection method that a line-type inspection method applied phased array ultrasonic testing and the area inspection method applied acoustic resonance method using developed moveable tapper. The acoustic signals were signal-processed and reproduced to a mapping image following the inspection area, and with the image, it was possible to determine the type of defect. Furthermore, an automated inspection system for within the CLP was proposed.

## 1. Introduction

**Figure 1:**
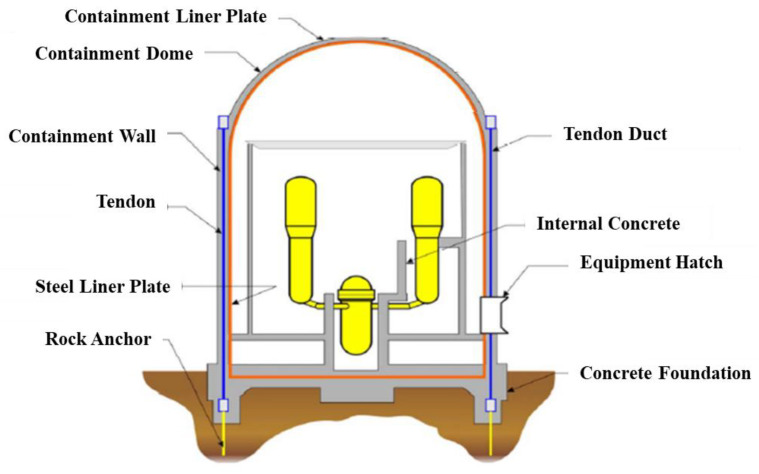
South Korean light-water type power reactor CLP structure [1].

The containment liner plate (CLP), as the final fourth level of a nuclear power plant protection wall, is a crucial structure that maintains the safety of a power plant by protecting the internal systems from the external environment and blocking radioactive materials from leaking into and out of the plant in the event of a nuclear accident. The CLP structure of the South Korean light-water-type power reactor is shown in Figure 1.

**Figure 2:**
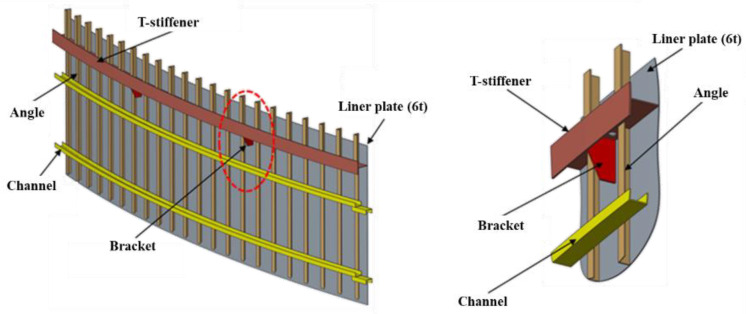
Liner plate structural connection [2].

Light-water-type power reactors, which account for most nuclear power plants in South Korea, have a cylindrical CLP, a sleeve foundation, and a dome ceiling cover. The cylindrical wall and hemispherical ceiling were constructed using reinforced concrete that was pre-stressed and post-tensioned. They contain liner plates that used carbon steel with a 6 mm nominal thickness for leak prevention and are structured with angles directly connected to the liner plates and channels that then connected the angles and brackets, as shown in Figure 2 [1,2,3,4,5,6].

After the detection of a CLP penetration defect owing to liner plate corrosion during the periodic inspection of the Hanbit Unit 2 in 2016, multiple other defects, such as corrosion, thickness decline, and rear side concrete voids were observed [7]. Concrete voids are unfilled parts that can be attributed to issues with the stiffener during casting. A corrosion-resistant passive film is generally applied to the CLP surface; however, as the voids do not contain an alkaline environment due to a lack of contact between the CLP and concrete, this film cannot be formed on voids. The residual oxygen and moisture within voids result in corrosion [8]. Liner plate corrosion reduces the plate thickness, which may seriously endanger the structural safety of nuclear power plants in South Korea [1,2,3,4].

Corrosion, thickness decline, and void defects on the rear side of a CLP cannot be visually inspected and detected. Thus, the thickness of the plate was measured by ultrasonic inspection to estimate the corrosion and thickness decline on the rear side.

Lee et al. [1] performed vibration signal analysis for void defects using a contact acceleration sensor. Kim et al. [5] analyzed the acoustic signal for the shape of the impact solenoid using a non-contact acoustic sensor. Peak et al. [9] conducted a study to inspection for CLP by ultrasonic testing. However, conventional ultrasonic thickness (UT), vibration measurement systems, and tapper devices are single-point-based inspection methods that only have a localized inspection area [1,5,9]. In addition, these methods incur substantial costs and time [10,11,12,13,14,15].

In this study, to address the limitations of the existing UT, vibration measurement system, and tapper device, a line scan was applied to the UT point method using a phased array ultrasonic testing (PAUT) method, and the wheels and solenoids were attached to the tapper to enable area inspection. Subsequently, the results of the A-scan signals were converted into images to develop a void-defect evaluation method.

## 2. Theory and Test Specimen Fabrication

### 2.1. Fabrication of CLP Mock-Up Specimen

The CLP mock-up test specimens were shell forms fabricated based on the structure of the nuclear power plant CLP, and three forms were created for the sound area, kissing bond, and void defects.

**Figure 3:**
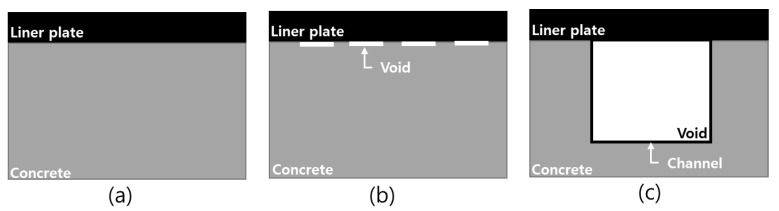
Three fabricated forms: (**a**) sound area, (**b**) kissing bond, and (**c**) void.

The sound area represents the state in which the liner plate and concrete are in complete contact and without defects. The kissing bond represents the state in which the liner plate and the concrete are in partial contact. The void represents the condition in which the liner plate and the concrete are completely separated from each other; this form was fabricated using an unfilled channel. The three fabricated forms are shown in Figure 3.

**Figure 4:**
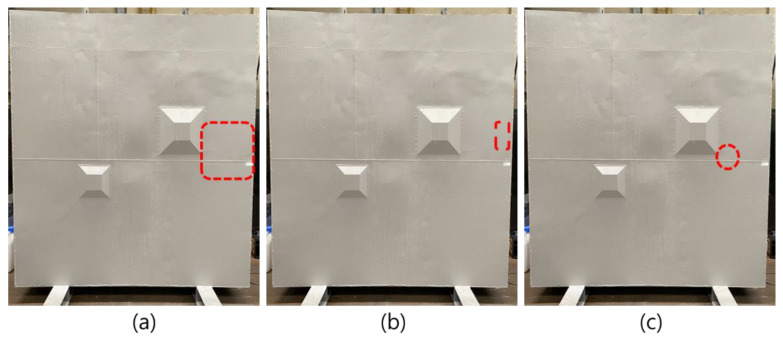
Fabricated CLP mock-up specimen: (**a**) inspection area, (**b**) kissing bond, and (**c**) void.

The specimen was a concrete structure with dimensions of 2400 × 3000 × 994 mm, consisting of a 6 mm carbon steel liner plate and an unfilled channel (void defect). The liner plate was surface-treated to prevent corrosion. Figure 4 shows a mock-up specimen of the fabricated CLP.

The unfilled channel had a “Π” shape, and the channel dimensions were 130 × 130 × 70 mm. The void defects are shown in Figure 3c and Figure 4b. Furthermore, Figure 4a shows inspection area using PAUT and ARM and (c) shows the kissing bond position.

### 2.2. Reflection Coefficient of PAUT

In the nuclear power plant CLP, the PAUT method with good focusing performance was used instead of the conventional UT method to reduce the void defect inspection time and improve inspection reliability [16]. The phased array ultrasonic transducer contacted the liner plate surface to focus ultrasonic waves between the liner plate and the concrete interface, ignored the ultrasonic waves transmitted into concrete, and comparatively analyzed the ultrasonic signals of sound area, kissing bond defect area, and void defects using reflection coefficients of the ultrasonic pulse-echo signal reflected from the interface. The reflection coefficient is shown in Equation (1) [17]:

(1) $$R_{p}=\frac{\rho_{2}c_{2}-\rho_{1}c_{1}}{\rho_{2}c_{2}+\rho_{1}c_{1}}=\frac{Z_{2}-Z_{1}}{Z_{1}+Z_{2}}$$

### 2.3. Principle of Acoustic Resonance Method

In the acoustic resonance method, an impact force was applied to a test specimen using an iron ball, hammer, and impact solenoid. The elastic wave generated from the impact was analyzed to identify the natural frequency depending on the form, dimensions, and constraints of the specimen. Two types of sensors were used to collect the elastic waves: contact accelerometer sensors and non-contact microphone sensors. A contact accelerometer sensor requires a contact medium and must be processed manually by an inspector, which can be time-consuming. In contrast, a non-contact microphone sensor does not require a contact medium. A fixing apparatus for the sensor is required; however, this has the advantage of automation through the fixing apparatus [18,19,20,21].

Moreover, the approach for determining the natural frequency depends on the form and characteristics of the specimen. The liner plate used as the specimen in this study was a thin plate with a thickness much smaller than its length and width. Therefore, the Kirchhoff–Love plate theory (Equation (2)) was used to determine the natural frequency of the liner plate [22].

(2) $$w_{mn}=\left(\frac{m^{2}}{a^{2}}+\frac{n^{2}}{b^{2}}\right)\sqrt{\frac{D\pi^{4}}{2\rho h}}$$

Here, *m* and *n* are the node numbers for different directions, *a* and *b* are the *x*-axis and *y*-axis widths of the carbon steel plate, respectively, *ρ* is the carbon steel plate density, *h* is the carbon steel plate thickness, and *D* is the bending stiffness of the plate, which can be calculated according to Equation (3).

(3) $$D=\frac{2h^{3}E}{3\left(1-\nu^{2}\right)}$$

E is the modulus of elasticity, and v is Poisson’s ratio. The Kirchhoff–Love plate theory is valid when the correlation between the thickness of the carbon steel plate and the *y*-axis dimension is h/b < 0.1 [23].

### 2.4. Experiment Equipment for Void Inspection

The equipment used in the PAUT experiment was an Olympus OmniScan MX2 of 16 ch, and 0.31 mm element pitch; the probe frequency was 5 MHz. The PAUT probe and equipment were focused on the bottom surface of the liner plate and set up to enable impedance change analysis at the interface between the liner plate and the concrete. The PAUT experiment data were analyzed using the Olympus Tomoviewer software.

ARM experiment was performed using GRAS acoustic sensor with a 10–20 kHz frequency collection and an NI-9250 (DAQ Board), LabVIEW data collection program. An impact solenoid was used as the impact source for the specimen, rather than an iron ball or hammer.

## 3. Movable Tapper Development for CLP

### 3.1. Establishing Inspection Conditions Using Finite Element Method (FEM) Simulation

**Figure 5:**
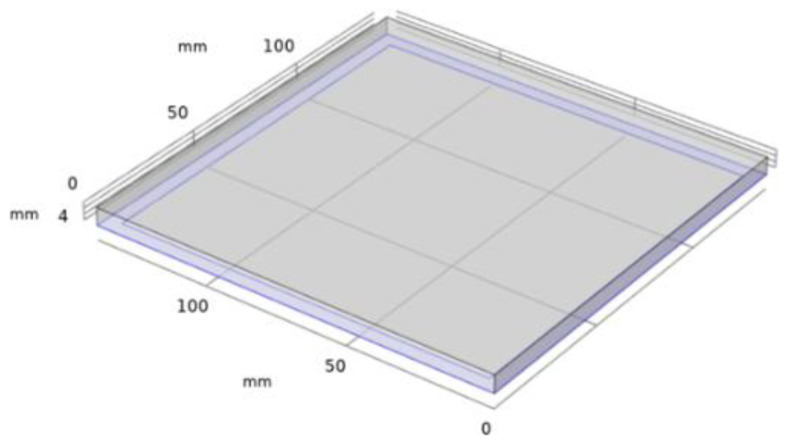
Liner plate modeling of FEM simulation in COMSOL.

In this experiment, an FEM simulation was implemented to select a sensor for collecting acoustic data, and the COMSOL Multiphysics program was used. The steel plate was modeled into a 6 mm thick 140 × 140 mm tetrahedral shape; a carbon steel material with a size of 130 × 130 mm was used to model the void defect in the mock-up specimen. The boundary conditions were equal to those of the shape in Figure 3c, and the actual specimen size to achieve the state in which the steel plate was attached to the concrete. Figure 5 shows the FEM simulation model produced by COMSOL. The liner plate was modeled, and the channel and concrete areas were set as fixed constraints and omitted from modeling. In addition, when an impact was applied, displacement of the carbon steel plate in the *x*- and *y*-axis directions was limited.

A square-wave impact was applied to the center of the model in Figure 5. The maximum force applied was 5 N, and the time of the sustained impact was 20 μs. The square-wave formula is given by Equation (4).

(4) $$f\left(t\right)=5*\left(1/1+\exp\left(-2*10^{7}\left(t-10^{6}\right)\right)\right)*2/\left(1+\exp\left(2*10^{-7}*\left(t-10^{-6}-2*10^{-5}\right)\right)\right)/4$$

**Figure 6:**
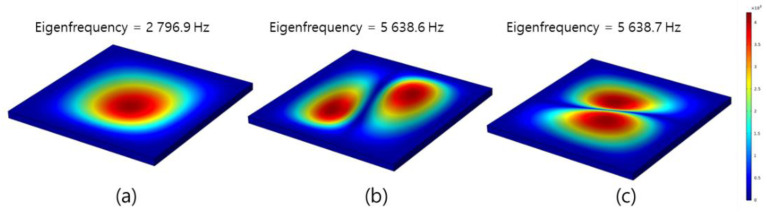
FEM simulation results for resonance frequency of liner plate: (**a**) first mode, (**b**) second mode, and (**c**) third mode.

The results of the linear plate resonance frequency in the FEM simulation are shown in Figure 6, where the frequencies of the first, second, and third modes were 2796.9, 5638.6, and 5638.7 Hz, respectively.

### 3.2. FEM Simulation Result Verification Experiment

**Figure 7:**
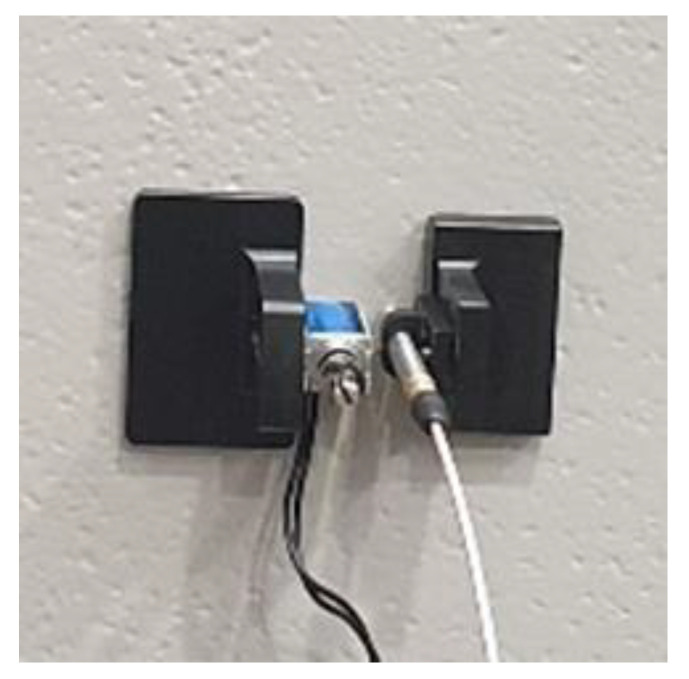
Experimental set up for FEM simulation result verification.

The FEM simulation results were experimentally verified. A jig was used to fix the solenoid impact source and acoustic sensor that collected signals from the CLP mock-up specimen void defect region, as shown in Figure 7. The distance between the acoustic sensor and the solenoid was 15 mm.

**Figure 8:**
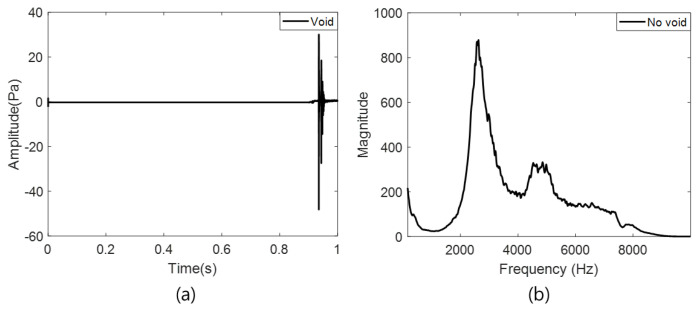
Verification experiment results for FEM simulation results; (**a**) A-scan signal and (**b**) frequency spectrum.

Figure 8 shows the FEM simulation verification experiment results. The A-scan signal collected by the acoustic sensor is shown in Figure 8a, whereas Figure 8b shows the frequency spectrum of the collected signal (a) through the Fast Fourier Transform (FFT). The results shown in Figure 8b confirm that the resonance frequency occurs close to 2.6 kHz, and that the second resonance frequency occurs at approximately 5 kHz. When comparing the FEM simulation results with the actual experimental results, the resonance frequency of the first mode had an error rate of approximately 8%. Therefore, the performance of the acoustic sensor that was selected based on the FEM simulation experiment was effective.

### 3.3. Movable Tapper Design Condition Establishment Using Experiments

Considerations for the movable tapper design are the impact height from the specimen surface to the solenoid, the signal collection distance between the acoustic sensor and solenoid, and the solenoid impact form.

**Figure 9:**
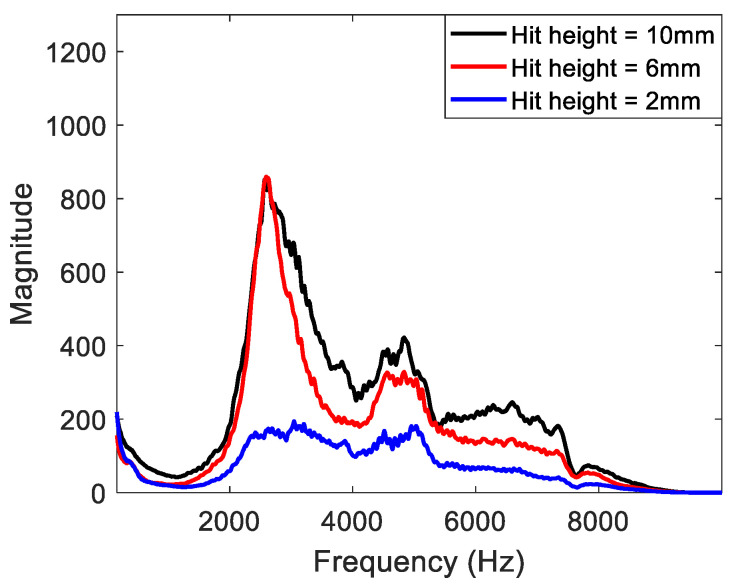
Experimental results for impact height.

The impact height was the solenoid stroke at 10 mm, and the height range from the specimen surface to the solenoid was 0–10 mm. Minimum, intermediate, and maximum impact heights of 2, 6, and 10 mm, respectively, were established for the experiment. The experimental results according to the impact height are shown in Figure 9 and were obtained from the frequency analysis of the collected A-scan signals. The resonance frequency could not be observed for an impact height of 2 mm because the impact load on the specimen surface was weak. When the impact height was greater than or equal to 6 mm, the resonance frequency could be clearly observed.

**Figure 10:**
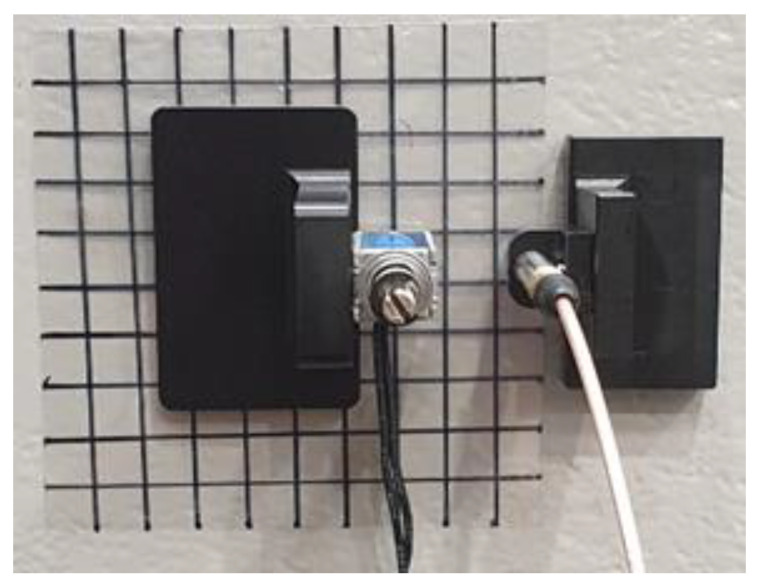
Experimental setup for data acquisition distance.

The experimental setup for the distance between the acoustic sensor and the solenoid is shown in Figure 10. A grid was utilized according to the collection distance. Because of the form of the fixing apparatus for the solenoid and the acoustic sensor, the established distances for the experiment were from 15 to 85 mm in 5 mm intervals.

**Figure 11:**
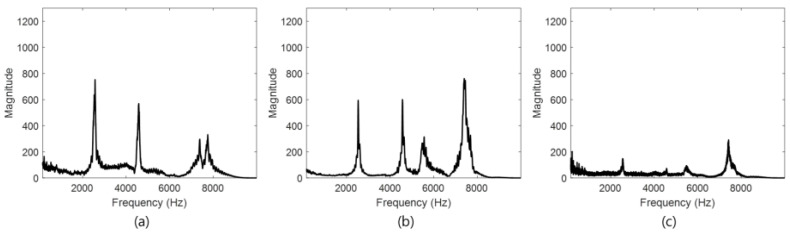
Experimental results for data acquisition distance; (**a**) 15 mm, (**b**) 70 mm, and (**c**) 85 mm.

Resonance frequencies were observed in the second mode until 70 mm between the acoustic sensor and the solenoid (Figure 11a,b). However, Figure 11c shows that the intensity of the resonance frequency decreased from 85 mm onward.

Based on the experimental results, the distance between the acoustic sensor and the solenoid was selected to be in the range of 15–70 mm. A suitable solenoid impact form was selected on the basis that no permanent damage could occur on the test object surface because the strength of the resonance frequency increased as the impact area decreased [4].

### 3.4. Design

**Figure 12:**
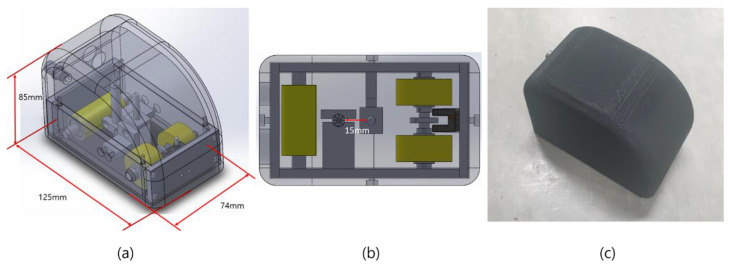
Movable tapper: (**a**) 3D modeling image isometric view, (**b**) bottom view, and (**c**) fabricated.

The movable tapper, designed using a 3D modeling program, is shown in Figure 12.

The movable tapper was designed using the variables acquired from the FEM simulation and the experimental results (Section 3.1, Section 3.2 and Section 3.3). The position of the solenoid impact was set to a height of 6 mm from the surface of the test object, and the distance between the acoustic sensor and the solenoid was set to 15 mm to minimize the size of the movable tapper. Additionally, a mechanism that allows for the solenoid to strike after every 25 mm of movement was developed.

Figure 12c shows the fabricated movable tapper. Internally, the tapper consisted of an acoustic sensor, a solenoid, and a drive unit. It was designed to be manually movable, and an external design was produced using a 3D printer.

## 4. Experiment Results for CLP Void

### 4.1. Experiment Results of PAUT

**Figure 13:**
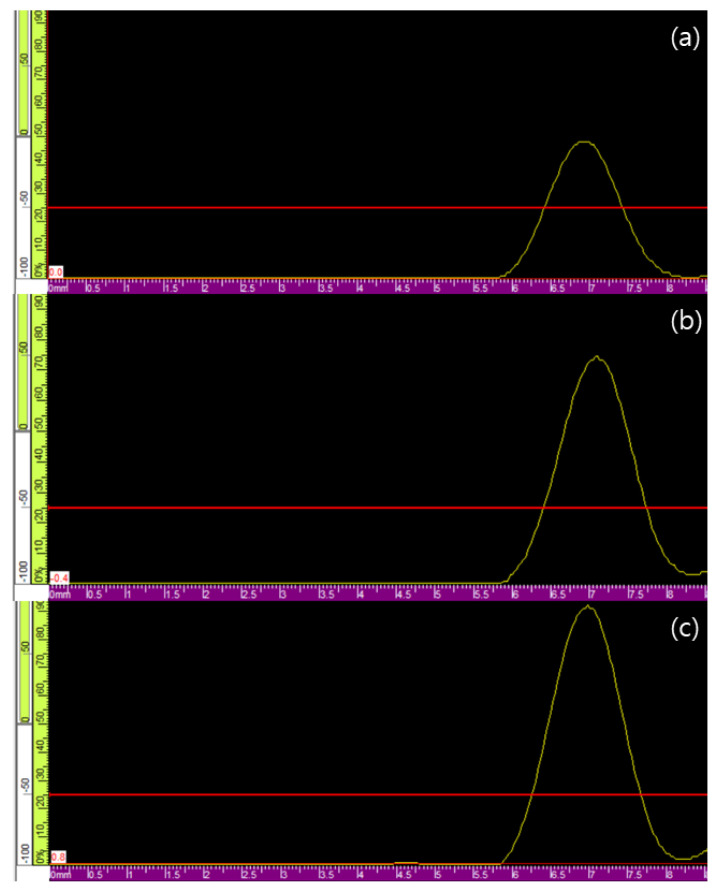
A-scan signal of PAUT for three defect types: (**a**) sound area, (**b**) kissing bond, and (**c**) void defect.

Figure 13 shows the results of the PAUT experiment, in which the A-scan signals of the (a) sound area, (b) kissing bond, and (c) void defect are shown.

Standardization was performed based on the impedance changes of the liner plate and concrete. The signal amplitudes were approximately 58%, 78%, and 91% in Figure 13a–c, respectively. However, a slight difference was observed in the roughness of the contact surface. The signal amplitude was reduced to half at the attachment between the liner plate and the concrete, and that from the kissing bond and void was reduced to 80–85%.

**Figure 14:**
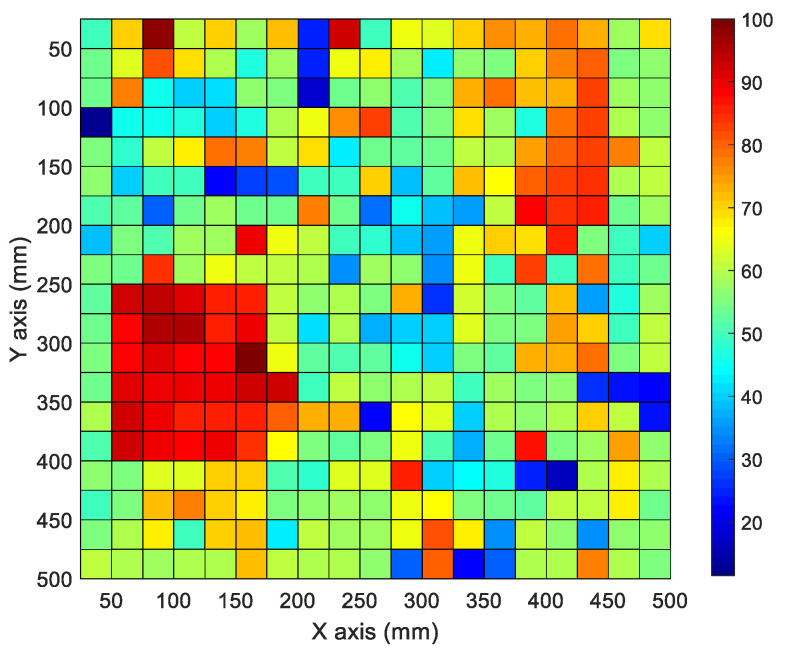
Signal mappings of PAUT for CLP specimen.

The mapped data of the inspected area in Figure 4a are shown in Figure 14. The collected A-scan signal amplitudes were measured at each point.

The PAUT signal impedance change occurred at similar positions in Figure 4b,c, which can be observed in the mapping data in Figure 14. The rapid signal decrease and increase in the mapping data were estimated to be errors from the contact medium.

### 4.2. Experimental Results of the Acoustic Resonance Method (ARM)

**Figure 15:**
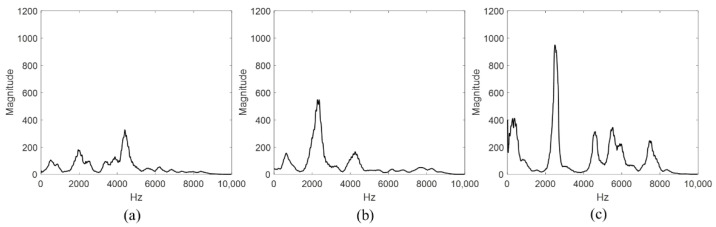
Frequency spectrum of ARM for three defect types: (**a**) sound area, (**b**) kissing bond, and (**c**) void.

The acoustic signals collected through the movable tapper were analyzed using signal processing techniques and FFT to represent the frequency spectrum of the CLP mock-up specimen by defect type, as shown in Figure 15.

The sound area signal is shown in Figure 15a, for which the maximum magnitude of the frequency spectrum was low at 150, and the liner plate void size was small when the plate and concrete were completely in contact. The signals collected from the kissing bond are shown in Figure 15b, for which the signal magnitude was approximately 600. The signals collected from the void are shown in Figure 15c, whereas in Figure 15b, the frequency signals collected from a large range of frequency bands are shown, and a magnitude of 950 was measured at 2.45 kHz. Moreover, the frequency spectrum was large at 5.5 and 7.5 kHz, and the frequency spectrum patterns are estimated as frequency traits from the simulated void.

**Figure 16:**
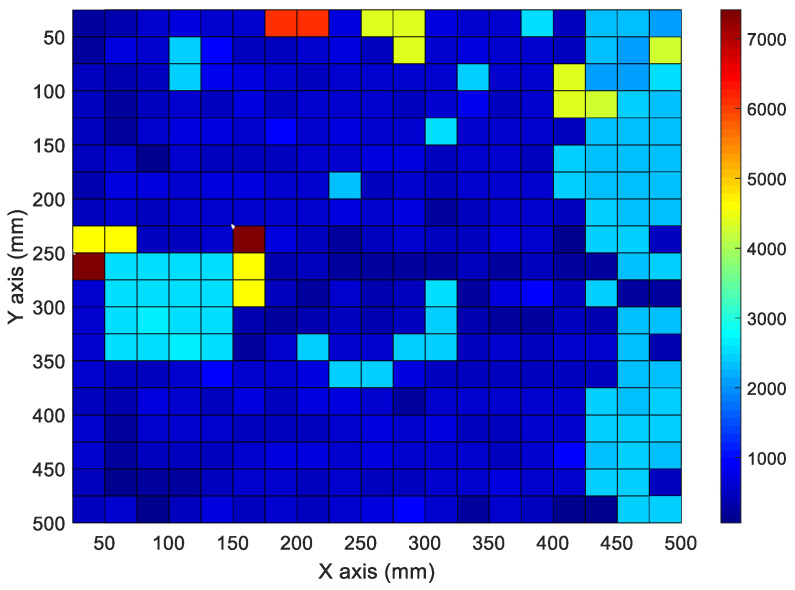
Signal mappings of frequency of maximum magnitude in the frequency spectrum for CLP specimen.

Figure 16 shows the mapping data from the inspection area of the CLP mock-up specimen using a movable tapper. A total of 2.45 kHz with the maximum magnitude in the frequency spectrum was used for the inspection area data mapping.

**Figure 17:**
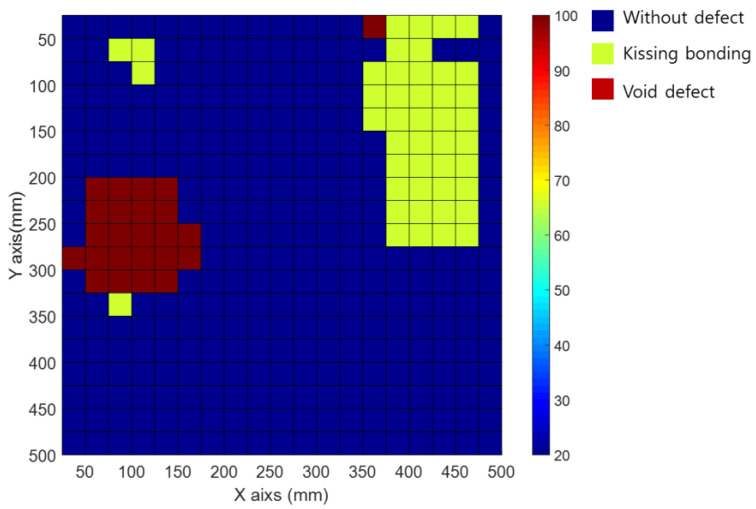
Signal mappings of frequency spectrum pattern for CLP specimen.

In the inspection area shown, according to the frequency with the maximum magnitude (Figure 16), the void and kissing bonds are collected in the same frequency band. The void and kissing bond areas can be distinguished from the sound area by utilizing the frequency with the maximum magnitude (Figure 16). However, they are difficult to distinguish from each other. The signals were mapped based on the frequency spectrum signal patterns rather than the frequency magnitude (Figure 16). The results of the analysis are presented in Figure 17.

The results shown in Figure 17 indicate that the signals at the void and kissing bond locations in Figure 4a,b, respectively, were successfully mapped. The defect type can be determined more accurately through the signal patterns of the frequency spectrum. In addition, they can be used to design an automated inspection system for the CLP.

## 5. Conclusions

In this study, conventional inspection methods, the UT and vibration measurement system, were enhanced by PAUT and a movable tapper to inspect void defects within the CLP. Each inspection method was presented with an area scan using a line-type scan and apparatus.

In line-type inspection using PAUT, the sound area could be distinguished, whereas the kissing bond and void defect were challenging to distinguish from each other. Additionally, signal errors frequently occurred owing to the contact medium, and it was confirmed that a quantitative signal collection based on uniform contact conditions was necessary.

The area inspection from the movable tapper with an applied ARM can be categorized into frequency spectrum magnitude analysis and frequency spectrum signal pattern analysis. From the frequency spectrum, the resonance frequency areas, for which the maximum magnitude value occurred in the kissing bond and void defect, were difficult to distinguish from each other. However, the frequency spectrum signal pattern analysis revealed varying patterns in the sound area, kissing bond, and void defect. 

In future studies, if an algorithm that automatically distinguishes between frequency spectrum signal patterns can be developed, an automated inspection system can be designed for the CLP based on the developed movable tapper and algorithm.

## Data Availability

The data that support the finding of this study are available from the corresponding author upon reasonable request.
